# Preoperative Arterial Embolization Facilitating Safe Resection of a Hypervascular Aggressive Angiomyxoma: A Case Report

**DOI:** 10.1002/ccr3.73667

**Published:** 2026-09-30

**Authors:** Yukako Takashima, Shinichi Terada, Yoshimichi Tanaka, Takashi Yamada, Kazuhiro Yamamoto, Yoshinobu Hirose, Tomohito Tanaka

**Affiliations:** ^1^ Department of Obstetrics and Gynecology Osaka Medical and Pharmaceutical University Takatsuki Japan; ^2^ Department of Pathology Osaka Medical and Pharmaceutical University Takatsuki Japan; ^3^ Department of Radiology Osaka Medical and Pharmaceutical University Takatsuki Japan

**Keywords:** aggressive angiomyxoma, hypervascular tumor, preoperative embolization, surgical resection

## Abstract

A 22‐year‐old woman presented with a 12‐cm hypervascular aggressive angiomyxoma. Difficult hemostasis during biopsy prompted preoperative selective arterial embolization followed by complete macroscopic resection with only 50 mL of blood loss. Preoperative embolization may facilitate safe resection of large hypervascular aggressive angiomyxomas.

## Key Clinical Message

In patients with large hypervascular aggressive angiomyxomas, preoperative selective arterial embolization may reduce intraoperative bleeding and facilitate safe and complete macroscopic resection. Assessment of tumor vascularity and feeding arteries may be helpful when significant bleeding is anticipated.

**Abbreviations**
AAM: aggressive angiomyxoma
ER: estrogen receptor
GnRH: Gonadotropin‐releasing hormone
PR: progesterone receptor
SMA: smooth muscle actin

## 1 Introduction

Aggressive angiomyxoma (AAM), a particularly rare mesenchymal tumor first described in 1983, occurs predominantly in women and most commonly arises in the genital tract and perineal–pelvic region [1, 2]. Although histologically benign, it exhibits local infiltrative growth and a high rate of local recurrence. Therefore, complete surgical excision remains the mainstay treatment [3]. Since significant intraoperative bleeding may hinder complete tumor resection, careful preoperative management is essential to ensure safe tumor removal. Although arterial embolization has occasionally been described for the management of AAM, evidence regarding its role as a planned preoperative strategy for markedly hypervascular tumors remains limited. We report the case of a large hypervascular AAM in which difficult hemostasis during biopsy prompted selective angiography and embolization, enabling complete macroscopic resection with minimal blood loss.

## 2 Case History/Examination

A 22‐year‐old, nulliparous Japanese woman presented with irregular genital bleeding. The patient had no relevant medical history. Physical examination revealed a soft, elastic mass extending from the left buttock to the perineal region. Laboratory tests showed a serum carbohydrate antigen 125 level of 23.8 U/mL (reference range, < 38.0 U/mL) and a carbohydrate antigen 19–9 level of 8.7 U/mL (reference range, < 37.0 U/mL).

## 3 Methods

### 3.1 Differential Diagnosis, Investigations

Ultrasonography revealed a 12‐cm well‐defined hypervascular tumor with a laminated appearance, whereas the uterus and bilateral ovaries appeared normal. Magnetic resonance imaging revealed a mass with isointensity on T1‐weighted images and mixed high and low signal intensities with a swirling appearance on T2‐weighted images, along with heterogeneous enhancement after contrast administration (Figure 1). 18F‐Fluorodeoxyglucose positron emission tomography/computed tomography showed increased 18F‐fluorodeoxyglucose uptake in the tumor. The results of a tumor biopsy were indicative of AAM.

**FIGURE 1:**
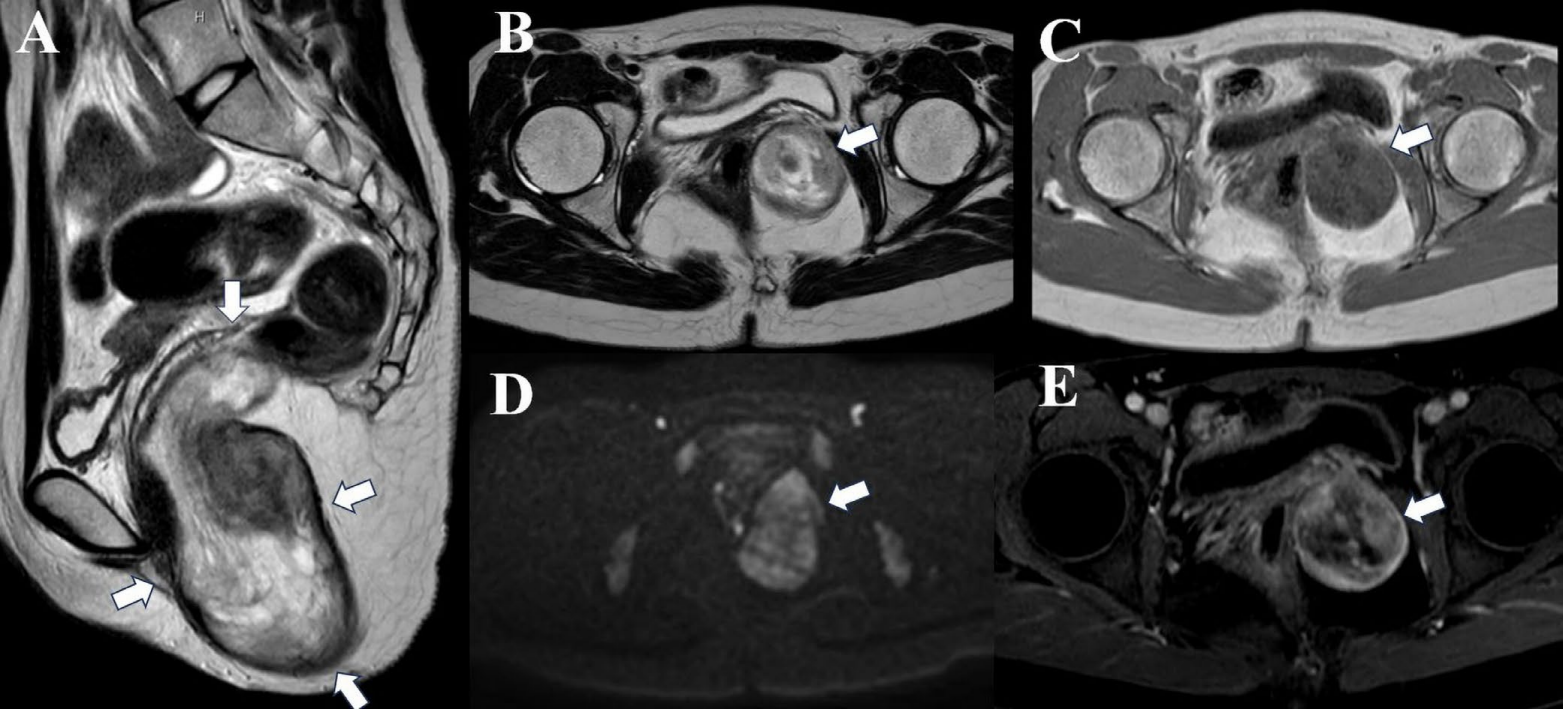
Magnetic resonance imaging of the pelvis. (A–C) T2‐weighted images showing a 12‐cm tumor with mixed high and low signal intensities and a characteristic swirling appearance (white arrow). The tumor showed a low signal intensity on T1‐weighted images. (D, E) High signal intensity on diffusion‐weighted images of the tumor and heterogeneous enhancement after contrast administration (white arrows).

### 3.2 Treatment

Surgical resection was planned as the definitive treatment. Oral relugolix, a GnRH antagonist, was administered at a dose of 40 mg once daily during the approximately 1‐month waiting period before surgery with the aim of reducing the tumor size as much as possible. No significant change in tumor size was observed during this period, and surgery was performed as planned. Because hemostasis was difficult to achieve during biopsy owing to the hypervascular nature of the tumor, preoperative arterial embolization was planned. The right femoral artery was accessed, and angiography demonstrated tumor‐feeding vessels arising from the left internal pudendal artery. The distal branches of the left internal pudendal artery were embolized using four Target XL 360 Soft Detachable Coils (2 mm × 6 cm), six coils (3 mm × 9 cm), and two coils (4 mm × 12 cm) (Stryker). Subsequent angiography of the superior and inferior gluteal arteries demonstrated additional tumor‐feeding vessels arising from branches of the inferior gluteal artery. These branches were embolized using four Target XL 360 Soft Detachable Coils (2 mm × 3 cm) and two coils (2 mm × 6 cm). Finally, left external iliac angiography was performed, confirming the absence of additional tumor‐feeding vessels. Repeat angiography confirmed near‐complete disappearance of tumor blood flow from the internal pudendal and inferior gluteal arteries (Figure 2). No complications related to the embolization procedure were observed. To minimize intraoperative blood loss by taking advantage of the reduction in tumor blood flow achieved by embolization, surgery was initiated approximately 1.5 h after completion of the embolization procedure. The surgery was performed with the patient in the lithotomy position. An approximately 10‐cm skin incision was made from the perineal region toward the left buttock. The tumor was circumferentially dissected from the surrounding normal tissue and excised. After tumor excision, no gross residual tumor was identified; therefore, complete macroscopic resection was considered to have been achieved. The operative time was 163 min, and intraoperative blood loss was estimated by the gauze‐count method and was 50 mL. Gross examination revealed that the tumor had a soft, smooth surface, whereas the cut surface was gelatinous (Figure 3). Histologically, it consisted of spindle cells with mild atypia embedded in a myxoid stroma rich in blood vessels. Immunohistochemically, the tumor cells were positive for estrogen receptor (ER), progesterone receptor (PR), smooth muscle actin (SMA), and desmin, and negative for CD34, with a Ki‐67 labeling index of 1% (Figure 4). An Olympus BX43 microscope and a Nikon Digital Sight DS‐5 M imaging system were used; no software was used for image adjustment. The final pathological diagnosis was AAM. Complete macroscopic resection was achieved; however, because the tumor was excised without an additional margin of surrounding normal tissue, the pathological surgical margin was positive (R1).

**FIGURE 2:**
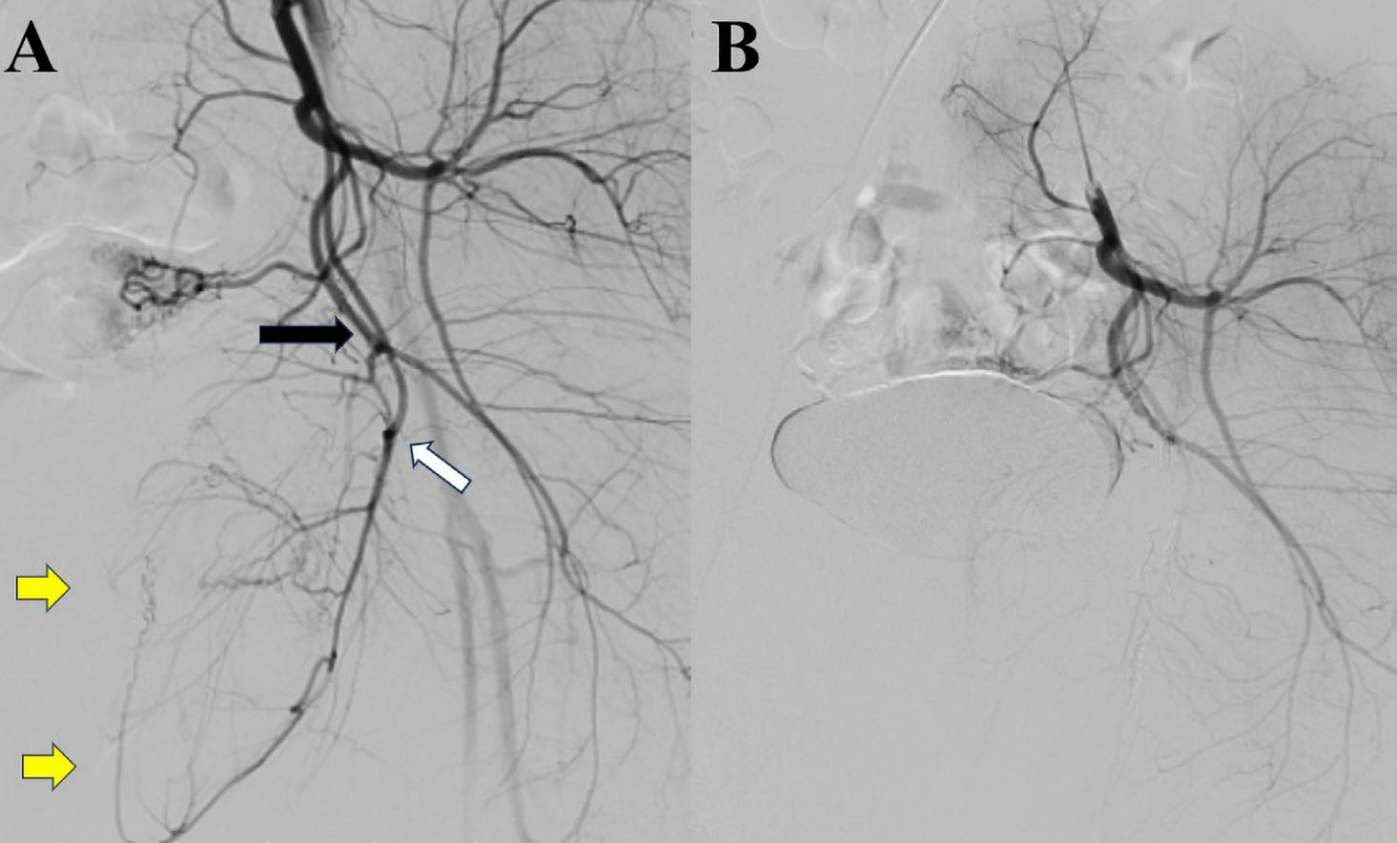
Angiographic findings. (A) Angiography showing that the tumor (yellow arrow) was supplied by the inferior gluteal artery (black arrow) and internal pudendal artery (white arrow). (B) After arterial embolization using detachable coils, angiography showed near‐complete disappearance of tumor blood flow from these feeding arteries.

**FIGURE 3:**
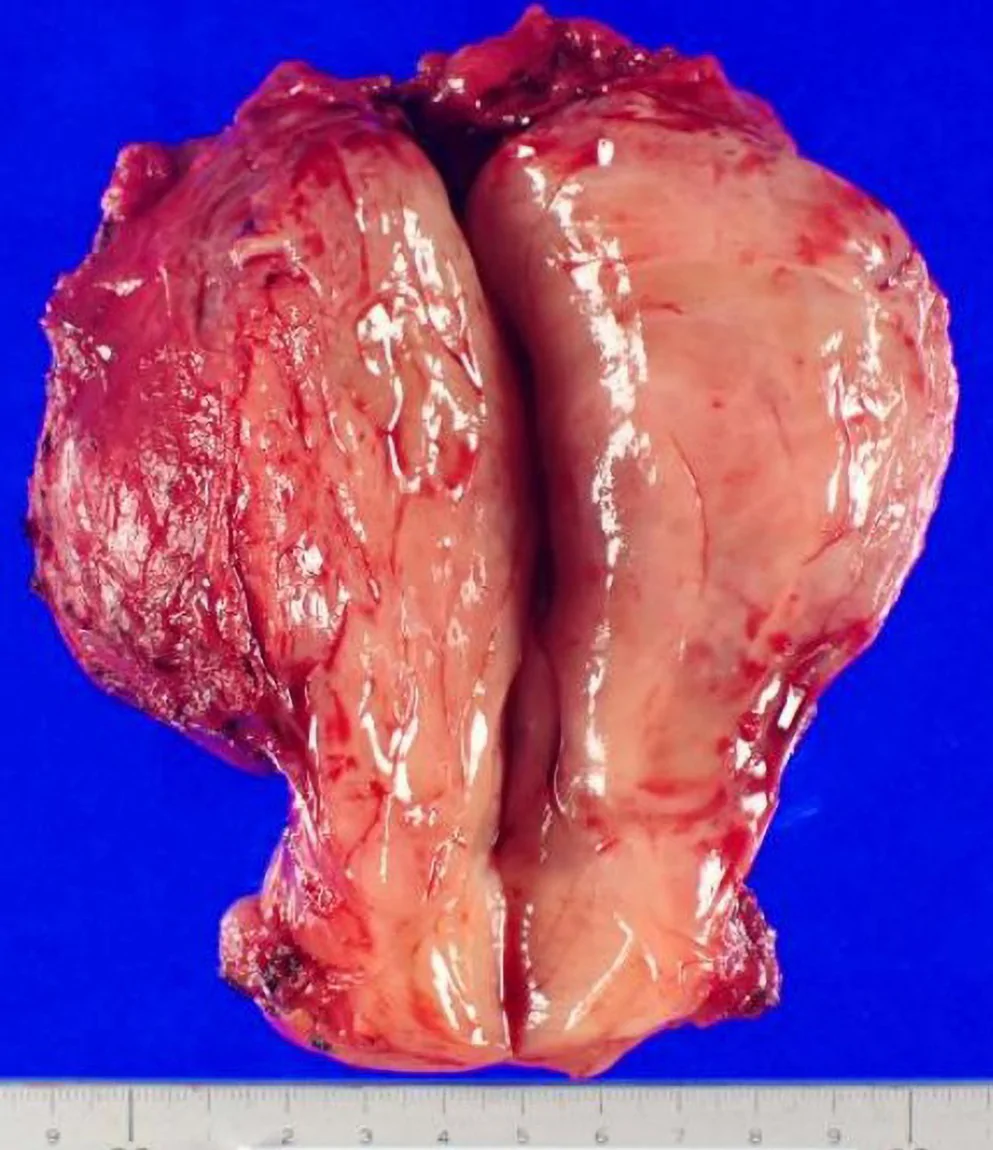
Gross appearance of the resected specimen. The tumor was soft with a smooth surface, and the cut surface was gelatinous.

**FIGURE 4:**
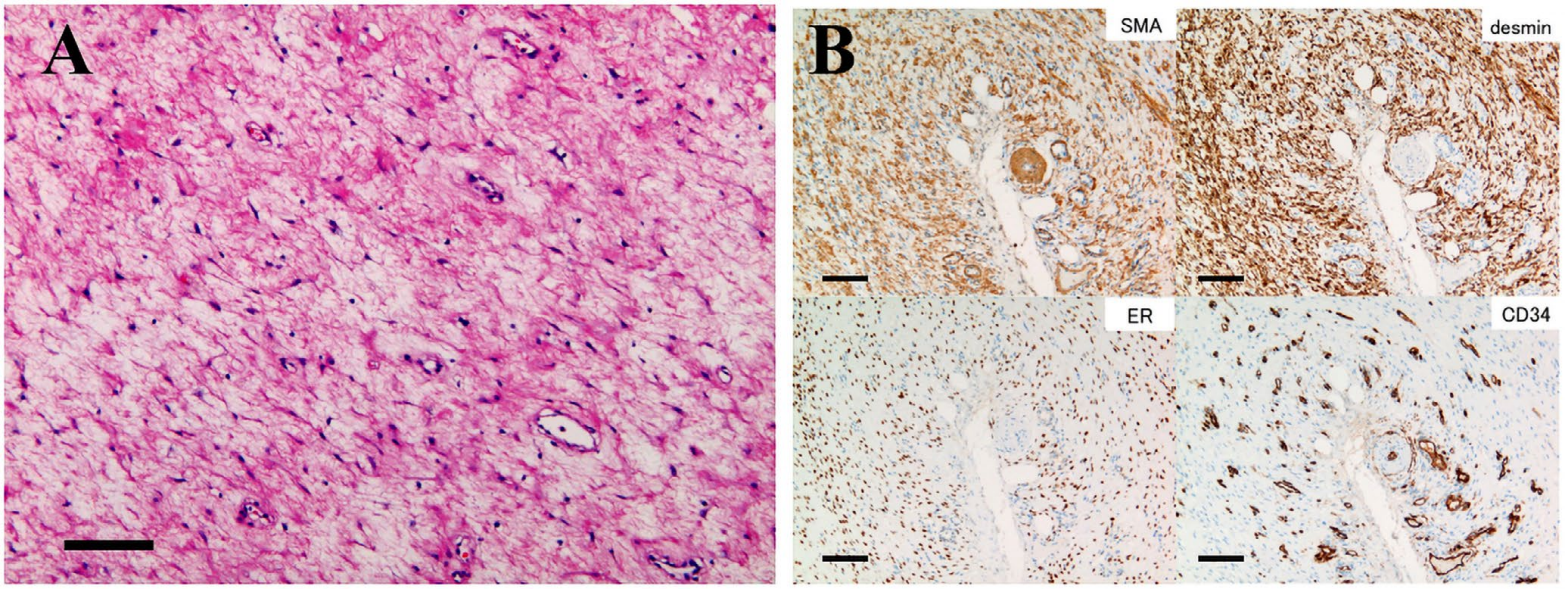
Histopathological findings of the tumor. (A) The tumor consisted of spindle‐shaped cells with mild atypia embedded in a myxoid stroma containing abundant blood vessels (H&E). Scale bar = 100 μm. (B) The tumor cells were positive for smooth muscle actin (SMA), desmin, and estrogen receptor (ER) and negative for CD34. Scale bar = 100 μm.

## 4 Results (Outcome and Follow‐Up)

The patient was followed every 6 months after surgery. Pelvic MRI performed at 9 months postoperatively showed no evidence of recurrence, and no clinical evidence of recurrence was observed at the 18‐month follow‐up.

## 5 Discussion

AAM is a rare soft tissue tumor that was first described in 1983 and is currently classified as a benign tumor of uncertain differentiation according to the World Health Organization classification. AAM predominantly occurs in women of reproductive age and typically arises in the pelvic and perineal regions. Most patients are asymptomatic or present with local pain, with tumors often > 4 cm in size at diagnosis [2].

On ultrasonography, AAM typically appears as a relatively well‐defined, irregularly shaped mass with hypoechoic features and internal vascularity [4]. On magnetic resonance imaging, it shows mixed high and low signal intensity on T2‐weighted images, with layered, swirling hypointense bands producing a characteristic “swirled” appearance. Gradual progressive enhancement after contrast administration is also commonly observed [2].

Histologically, AAM is characterized by low cellularity, with small, oval, spindle‐shaped, and stellate cells embedded in a loose myxoid stroma containing delicate collagen fibers and numerous medium‐sized blood vessels. Immunohistochemically, tumor cells are typically positive for desmin, SMA, ER, and PR, and may show CD34 reactivity. The Ki‐67 proliferation index is usually low [2, 5]. These histological and immunohistochemical findings are consistent with those observed in the present case.

Surgical excision is the recommended treatment of AAM. AAM can also occur during pregnancy. Although surgical resection is generally considered the primary treatment, management should be individualized according to each case. Vaginal delivery may also be feasible [6]. However, the reported recurrence rate is 30%–50% owing to its locally aggressive nature, with most recurrences occurring within 3 years [2, 3, 7, 8]. Recurrence has been reported regardless of whether the resection margins are negative or positive [7]. Therefore, although surgical resection remains the mainstay of treatment, achieving a safe and precise resection is equally important. Since many AAMs express ER and PR, hormonal therapy, such as GnRH antagonists, has been explored as a treatment option alongside surgery. Hormonal therapy may reduce tumor size, thereby facilitating complete resection or helping control recurrent disease [3, 9]. In the present case, a GnRH antagonist was administered during the approximately 1‐month waiting period before surgery with the aim of reducing the tumor size as much as possible; however, no substantial reduction in tumor size was observed.

Because tumors with abundant vascularity may lead to significant intraoperative bleeding, surgical resection of AAM can result in considerable blood loss [7]. Preoperative embolization has been reported to be a useful approach to reduce intraoperative bleeding in various tumors [10, 11, 12, 13], including gynecological tumors. Studies have reported that preoperative embolization for vaginal tumors is effective in reducing intraoperative bleeding [14, 15]. Although few reports have described the use of embolization for AAM, tumor ischemia induced by this procedure has been suggested to improve lesion visibility during surgery [16]. Lara et al. [17] have reported one case of preoperative arterial embolization among 32 cases of pelvic AAM in which R0 resection was successful using this approach. Furthermore, selective angiography can help navigate the vascular anatomy and guide the surgical approach [18]. For tumors requiring extensive resection, or when preservation of reproductive function is important, preoperative embolization may reduce the risk of surgical complications. In our case, hemostasis was difficult to achieve during the biopsy, indicating marked tumor vascularity. Angiography revealed that the tumor was mainly supplied by the branches of the internal pudendal and inferior gluteal arteries, which were embolized preoperatively. This approach may have contributed to successful tumor resection with minimal intraoperative blood loss. Although reports on its use are limited, these findings highlight the potential of preoperative embolization in AAM, especially for large or hypervascular tumors.

## 6 Conclusion

In conclusion, we report a rare case of AAM that was successfully treated with preoperative arterial embolization followed by surgical resection. Although the efficacy of preoperative embolization cannot be established from a single case, it may be considered as a potential strategy to reduce intraoperative bleeding and facilitate safe tumor resection in selected patients with large, markedly hypervascular AAMs. Further accumulation of cases is needed to clarify its clinical utility. Since AAM can recur even after long periods of remission and given that the follow‐up period in the present case was only one and a half years, long‐term follow‐up will be necessary in future studies.

## Author Contributions

**Yukako Takashima:** investigation, writing – original draft. **Shinichi Terada:** writing – review and editing, writing – original draft, supervision, investigation, conceptualization. **Yoshimichi Tanaka:** investigation, writing – review and editing, methodology. **Takashi Yamada:** investigation, writing – review and editing. **Kazuhiro Yamamoto:** investigation, methodology, writing – review and editing. **Yoshinobu Hirose:** investigation, writing – review and editing. **Tomohito Tanaka:** supervision, writing – review and editing, validation.

## Funding

The authors have nothing to report.

## Ethics Statement

This study was approved by the Ethics Committee of Osaka Medical and Pharmaceutical University.

## Consent

Written informed consent was obtained from the patient for the publication of this case report and the accompanying images. A copy of the written consent form is available for review by the journal's Editor‐in‐Chief.

## Conflicts of Interest

The authors declare no conflicts of interest.

## Data Availability

The data that support the findings of this study are available from the corresponding author upon reasonable request.
